# From Modern Planetary Health to Decolonial Promotion of One Health of Peripheries

**DOI:** 10.3389/fpubh.2021.637897

**Published:** 2021-06-10

**Authors:** Oswaldo Santos Baquero, Mario Nestor Benavidez Fernández, Myriam Acero Aguilar

**Affiliations:** ^1^Department of Preventive Veterinary Medicine and Animal Health, School of Veterinary Medicine and Animal Science, University of São Paulo, São Paulo, Brazil; ^2^nPeriferias – Research Group on Peripheries, Institute of Advanced Studies, University of São Paulo, São Paulo, Brazil; ^3^Facultad de Humanidades y Ciencias de la Educación, Universidad de San Buenaventura, Bogotá, Colombia; ^4^Departamento de Salud Animal, Grupo de Investigación en Estudios Humano Animal, Facultad de Medicina Veterinaria y de Zootecnia, Universidad Nacional de Colombia, Bogotá, Colombia

**Keywords:** One Health of Peripheries, modernity, coloniality, decolonial turn, health inequities, One Health, Planetary Health, more-than-human biopolitics

## Abstract

The concept of Planetary Health has recently emerged in the global North as a concern with the global effects of degraded natural systems on human health. It calls for urgent and transformative actions. However, the problem and the call to solve it are far from new. Planetary health is a colonial approach that disregards alternative knowledge that over millennia have accumulated experiences of sustainable and holistic lifestyles. It reinforces the monolog of modernity without realizing that threats to “planetary health” reside precisely in its very approach. It insists on imposing its recipes on political, epistemological, and ontological peripheries created and maintained through coloniality. The Latin American decolonial turn has a long tradition in what could be called a “transformative action,” going beyond political and economic crises to face a more fundamental crisis of civilization. It deconstructs, with other decolonial movements, the fallacy of a dual world in which the global North produces epistemologies, while the rest only benefit from and apply those epistemologies. One Health of Peripheries is a field of praxis in which the health of multispecies collectives and the environment they comprise is experienced, understood, and transformed within symbolic and geographic peripheries, ensuing from marginalizing apparatuses. In the present article, we show how the decolonial promotion of One Health of Peripheries contributes to think and advance decentralized and plural practices to attend to glocal realities. We propose seven actions for such promotion.

## Introduction

Modernity is a popular concept, often referred to the idea of progress, to positive and necessary changes to build a better future. Less famous is the critical comprehension of the modernity/coloniality cultural complex. This is not fortuitous; modernity is a narrative built by Western civilization to highlight its achievements (rationality, science, and technology) and conceal its dark side (genocide, expropriation, forced displacement, and exploitation) (1–3).

This dark side of modernity is coloniality; it is “the underlying logic of the foundation and unfolding of Western civilization from Renaissance to today of which historical colonialisms have been a constituent, although, downlpayed dimension” (3). Colonialism designates the political, social, and cultural domination in territories occupied by Europeans, typical of the period of colonization of America, which, far from being the discovery of America, was what Dussel called the discovery of an invasion and framed as the very origin of modernity (4).

The global South is a metaphor regarding the “field of epistemic challenges that seek to repair the damage and historical impacts caused by capitalism in its colonial relationship with the world” [translation is ours] (5). Therefore, the global South also includes Northern places. With the epistemologies of the South, the critiques of modernity cease to be exclusively internal (from the global North), making the colonial aspects of modern rhetoric evident (6). The epistemologies of the South show us that beyond economic crises, dictatorships, and corrupt governments, we are experiencing a crisis of civilization of more than five centuries (7), with devastating effects on health.

In Latin America, philanthropic support has helped to mitigate some of the health effects of the crisis of civilization, transferring small fractions of the wealth of a few rich philanthrocapitalists to the poorest, without affecting the consumption and accumulation patterns of the former, and enabling major transformations in the material conditions of the latter. This has made it possible to legitimize the elites and avoid responsibility for the poverty they generate and the exploitation that underpins the growth of their wealth. The Rockefeller Foundation's philanthrocapitalism has been around since the early 20th century, with strategies to shape the health professions and structure public health services (8–10).

But such strategies have also generated decolonial health responses. This is the case of Collective Health (9, 11), Critical Epidemiology (12), and South-South International Health (13). However, these responses inherited part of the colonial anthropocentrism and have treated health as a predominantly human phenomenon. Other beings appear only as vectors, reservoirs, or determinants of human health. Notwithstanding, it is worth highlighting the progress of the Ecuadorian school in its debates on the social determination of animal health (14) and animal production management (15).

In the report of The Rockefeller Foundation–Lancet Commission on Planetary Health, non-human beings appear within terrestrial systems that only have instrumental value, due to their role in human health (16). However, animal health takes prominence in One Health, another approach fostered by the Foundation. One Health refers to the human-animal-environmental health interface and has gained popularity for its convenience to address pandemics, emerging diseases, and antimicrobial resistance.

Like previous projects of the Foundation, Planetary Health and One Health can be read as proposals for preserving the capitalist order in the face of the perceived need for social change. More specifically, these two approaches pursue the prevention and control of environmental deterioration and animal diseases that impact human health, to avoid more instability in the capitalist order. As might be expected, the colonial aspects of these proposals have not been unnoticed (17–19), and in this paper, we will contribute by further exploring those aspects.

One Health of Peripheries is a decolonial response to experience, understand, and improve the well-being of marginalized multispecies collectives (20). Baquero presents the biopolitics, social *determination*, and field of praxis of One Health of the Peripheries, highlighting the symbolic character of the peripheries and leaving implicit its decolonial foundation (20). One of the features that shows this foundation is the opposition to animalization, a marginalizing apparatus registering non-human animals and marginalized human groups in colonial domination spaces that determine epidemiological profiles.

The excess risks underlying peripheral epidemiological profiles increase the relevance of primary, secondary, and tertiary prevention, that is, of measures directed at specific factors to (1) avoid, (2) early detect and treat, and (3) mitigate the effects of diseases or ill-health. However, the preventive approach is limited to a negative ontology of health, to the absence of diseases or ill-health. On the other hand, health promotion works on a positive ontology, regarding health as a resource and capability to live well. Despite the overlap between prevention and promotion, as the first subsumes the second (environmental sanitation prevents diseases and not having diseases increases the resources and capabilities to live well), the absence of diseases or ill-health is not enough in terms of promotion because that absence does not exhaust the possibility of a better life. Promotion is not restricted to risk factors or specific problems; it also works on resources and capabilities.

One Health of Peripheries is inherently preventive because its field of praxis generates excess risk and disease burden. However, peripheries are more than collections of risks and injuries; they have structurally oppressed resources and capabilities, which the ecology of knowledge can release in a multispecies health framework. Such release is the task of decolonial promotion of One Health of Peripheries.

In what follows, we present the myth of modernity and then continue with the colonial precedents of the Rockefeller Foundation's philanthropy and the coloniality in the report of The Rockefeller Foundation–Lancet Commission on Planetary Health. After this decolonial turn, we move to the ecology of knowledge to frame our proposal of decolonial promotion of One Health of Peripheries. This paper is a continuation of another one dedicated to introducing One Health of Peripheries (20).

## Modernity and Coloniality

Modernity designates a political, social, and cultural European process that in the 15th century allowed the emergence of capitalism, and since then, its development as a global economic system (21). Modernity has as a backdrop the idea of unlimited progress. Economic and social changes promoted by scientific and technological development promised the construction of a better future (22). The Eurocentric and colonial character of modernity has been questioned, particularly by the Latin American decolonial turn (23–25). Dussel pointed out two connotations of modernity: one, primary and positive, that understands modernity as an effort of rational emancipation that opens for humanity a new historical development, and the other, secondary and negative, in which modernity justifies irrational violence (26). According to this perspective, the only civilizing possibility for the “barbarian” peoples seems to be their gradual incorporation into the modern and Eurocentric project that depends to a large extent on the epistemological authority and alleged ontological superiority (racial, ethnic, geopolitical) of the global North (2). The incorporation to that project (modernization) has not been, however, an encounter between equals, but on the contrary, a violent conversion.

This violence, invested with heroism and redemption, marks the myth of modernity, synthesized by Dussel in seven elements: ‘(1) Modern (European) civilization understands itself as the most developed, the superior, civilization. (2) This sense of superiority obliges it, in the form of a categorical imperative, as it were, to “develop” (civilize, uplift, educate) the more primitive, barbarous, underdeveloped civilizations. (3) The path of such development should be that followed by Europe in its own development out of antiquity and the Middle Ages. (4) Where the barbarian or the primitive opposes the civilizing process, the praxis of modernity must, in the last instance, have recourse to the violence necessary to remove the obstacles to modernization. (5) This violence, which produces, in many different ways, victims, takes on an almost ritualistic character: the civilizing hero invests his victims (the colonized, the slave, the woman, the ecological destruction of the earth, etc.) with the character of being participants in a process of redemptive sacrifice. (6) From the point of view of modernity, the barbarian or primitive is in a state of guilt (for, among other things, opposing the civilizing process). This allows modernity to present itself not only as innocent but also as a force that will emancipate or redeem its victims from their guilt. (7) Given this “civilizing” and redemptive character of modernity, the suffering and sacrifices (the costs) of modernization imposed on “immature” peoples, enslaved races, the “weaker” sex, etcetera, are inevitable and necessary' (1).

Such suffering and sacrifice become less visible in the light of the seduction that turns modernity into aspiration, rather than imposing it through systematic and constant repression: ‘colonizers also imposed a mystified image of their own patterns of producing knowledge and meaning. At first, they placed these patterns far out of reach of the dominated. Later, they taught them in a partial and selective way, in order to co-opt some of the dominated into their own power institutions. Then European culture was made seductive: it gave access to power. After all, beyond repression, the main instrument of all power is its seduction. Cultural Europeanisation was transformed into an aspiration. It was a way of participating and later to reach the same material benefits and the same power as the Europeans: viz, to conquer nature in short for “development.” European culture became a universal cultural model' (2). But not everyone attains the aspiration. The ontological superiority of the myth of modernity limits material benefits and the exercise of power so that racial, ethnic, and geocultural attributes frustrate or advance the aspiration, depending on their configuration (27).

The configurations of these attributes define the place of hegemonic production of epistemologies of health and, what is more important, how they materialize in health. Within modernity, the global North produces epistemologies. In contrast, the global South is limited to benefit from the transfer of knowledge or knowledge building within the epistemological production patterns established by modernity. As we will see, the global North's health discourses align with the interests of the dominant groups of dominant nations, and to the extent that they neglect the interests of peripheral groups, they induce particular epidemiological profiles.

## Colonial Precedents of The Rockefeller Foundation

Capitalism, made possible by coloniality, generates figures like the one recently reported by Coffey and collaborators (28): in 2019, the world's billionaires, just 2,153 people, accumulated more wealth than 4.6 billion people. In other words, in a world population of 7.7 billion, the wealth concentrated by 0.000028% of the population was greater than that of 59.7%. In light of the so-called Law of diminishing marginal utility, figures like that make possible the transfer of small fractions of wealth from the wealthiest to the poorest without affecting the former's consumption and accumulation patterns while enabling major transformations in the material conditions of the latter. On the one hand, we can see these transformations as philanthropic successes. On the other hand, as a strategy to legitimize the elites and avoid responsibility for the poverty they generate and the exploitation that underpins the growth of their wealth. The dialectics between both sides reproduces inequalities and determine conditions of possibility to produce alternatives.

The mentioned transfers can increase the symbolic and cultural capital of elites and consequently their economic capital. Moreover, legitimization strategies are also economic investments. Among the main strategies is the influence on the educational system to favor the reproduction of the dominant classes by forming profiles to occupy high positions in the state bureaucracy and the field of power (9). It was not by chance that in the early 20th century, the United States' industrialization allowed the accumulation of great fortunes and the establishment of influential universities (today leading prestigious global rankings according to modern criteria), many of which are partially homonyms with their founders' magnates (29). John D. Rockefeller, the first world billionaire and owner of the Standard Oil Company, contributed to founding the University of Chicago (29).

According to Vieria-da-Silva (9), philanthropists at the beginning of the 20th century anticipated a social reform that they saw as inevitable, investing in scientific approaches to social issues that did not threaten the capitalist order. The Rockefeller Sanitary Commission was created in 1909 (The Rockefeller Institute in 1901 and The Rockefeller Foundation in 1913). One of its central objectives was the industrialization of the agrarian South and its articulation to the capitalist interests of the North (9).

The Rockefeller Foundation continued to invest and intervene in the research and development of medicine (9). In 1947, its official Fred Sopper became director of the Opas, an institution subordinated to the United States' health policies and officially directed from that country until 1958 (9). Only after the Second War, with the creation of the WHO, the Opas became a Regional Office of that organization. During the Cold War, the United States' foreign policy, in defense of free trade and foreign investment, involved the creation of a favorable image (9). According to Tota, Nelson Rockefeller, Coordinator of the Office of the Coordinator of the Inter-Americans Affairs, contributed to an explicit project to promote the United States' image (30).

In the dispute over the monopoly of legitimate healthcare practices in Latin America, the Rockefeller Foundation's goal was to replace the French model (9). In the 1950's, the Opas was fundamental to this objective, through its strategies to spread Preventive Medicine, an ideological movement to protect the interests of Private Medicine, in the face of two problems: the increasing cost of Medical Care in the United States and the possibility of a State intervention (8). These problems already worried the American Associations of medical colleges, as Arouca showed (8) by citing Fishbein and Bierring (31): “There is a special need that the medical profession develops some method by which the greatest possibilities of modern medicine in the way of diagnosis, treatment and prevention of diseases, may be brought within the reach of all people. This function, it is *believed, should be performed by the medical profession and not to any form of State Medicine*” [the original citation is entirely emphasized].

## Coloniality In The Report of The Rockefeller Foundation–Lancet Commission on Planetary Health

The report of the Rockefeller Foundation-Lancet Commission on Planetary Health maintains the Foundation's historical concern with inequality, the health of the poorest, and the environment. Such insistence is again a colonial proposal for preserving the capitalist order in the face of the perceived need to avoid environmental deterioration and its impacts on human health.

Although we discussed some Rockefeller Foundation's colonial precedents in the previous section, it is worth noting that Planetary Health also has precedents omitted in the report (19). Over the last half-century, integrative medicine, holistic medicine, and many scholars have talked about the need for a healthy planet, even using the expression “planetary health” (19). So in what follows, we continue previous contributions that look beyond the *ahistorical* and colonial perspective of the Rockefeller Foundation-Lancet Commission report (19, 32, 33).

In a typically colonial attitude, the report ignores an enormous diversity of worldviews that do not separate humans from nature or think that degrading nature does not affect human health and well-being. Worldviews with millenary legacies in which it is not new to think that health and well-being are also nature: “The importance of the natural environment in supporting human health and well-being *is only becoming clear* as the Earth's systems are degraded” [emphasis is ours] (16).

How is the modern trajectory of humanity progressing if there have never been so many victims of genocide, dispossession, forced displacement, and exploitation? Which humanity is the one that progresses? The same colonizing and modern humanity for which there is only one civilization, even though Western civilization is known to coexist with other civilizations: “Put simply, planetary health is the health of human civilization and the state of the natural systems on which it depends” (16).

It is not the humanity of backward and irrational peoples with visionary healers. It is rational humanity with visionaries from the global North: “[…] Tony McMichael whose visionary book Planetary Overload, published more than 20 years ago, presciently addressed many of the issues that confront the world at present” (16).

The previous decolonial reading of the report does not imply its total rejection. The problems pointed out by the Commission must be solved, and it is pertinent to evaluate the attempts at resolution, for which, as the Commission indicates, quantitative indicators are helpful. But these should be used considering their different implications.

Let's look at two examples from the report. Between 1990 and 2012, the percentage of stunted children decreased from 50 to 30%, a significant advance in relative terms. However, in absolute terms, this reduction represented an increase of 14 million children, a number only exceeded by the total population of 7 European Union member states in 2011. Overall, there were 58 million children—predominantly from the global South—stunted in 2012, a number surpassed only by the population size of 4 European Union member states in 2011. Thus, in its absolute and relative version, a numerical indicator tells different stories that must be considered in a critical and integrated way. In the global South, it is no progress to have millions of additional stunted children, while wealth concentration in the global North continues. Moreover, relative indicators fuel a discourse of hope, of the possibility of unlimited “progress,” causing increasingly smaller proportional damages.

The other example regards reducing the percentage of people in extreme poverty during the last two centuries. This reduction represents important improvements in the well-being of those who come out of extreme poverty. However, when the threshold is USD$1.9/day, those who survive on USD$2/day are not in extreme poverty. Who decides that surviving on USD$2/day (or on USD$50/day, in a state of frequent frustration at trying to satisfy manufactured consumer needs) is not a state of extreme poverty? Analyzing thresholds together with the underlying distribution allows comparison all individuals of the population. Otherwise, only mentioning the reduction in the fraction or number of individuals within unfavorable categories may conceal that changes occur in intervals far from thresholds that decision-makers would accept for themselves in the global North.

Thresholds help to identify limits from which damages become irreversible: “Action has to be taken before irreversible changes in key Earth systems occur, which will require decision-making under uncertainty (panel 13) about the critical thresholds or rates of deterioration of these systems” (16). In this sense of warning of catastrophes, thresholds are helpful to raise awareness and generate changes. However, at the same time, they can promote policies of acceptable minimums to avoid only irreversible changes instead of promoting multispecies flourishing.

Policies of acceptable minimums are symptomatic of crisis that leads not to crossing or scarcely crossing minimum thresholds. A crisis that, when it becomes persistent, ceases to be explained and becomes an explanation (34). Thus, a previous level of causality that perpetuates the *status quo* is lost. This is evident in the report, in its fragmented descriptions of the threats to Planetary Health. For example, it presents changes in land use as a human action on the “environment”, with deleterious effects on health, without considering their causes (16). From the perspective of One Health of Peripheries, the problem to solve resides in the capitalization of land that generates forced displacement of millions of people and animals, reduces biodiversity, and worsens the climate crisis.

The report identifies several causes of deterioration in health, and we agree with that identification. Our critique here is about the omission of previous causal levels. We also agree with the report in other points: the inconvenience of GDP as a measure of progress; technological improvements are not sufficient to reduce the environmental footprint because they can stimulate consumption and increase the footprint (rebound effect); governance transformations are necessary. We agree with a good part of the key messages and the conclusions. Our disagreement is, as shown by the previous decolonial viewpoint, in the interpretation of these messages and in the premises of the conclusions. From our reading, the report's proposal is convenient to preserve the *status quo* that makes the health of the planet ill.

The report calls for price stability and malnutrition management to fight hunger but not for food sovereignty and security: “[E]nsure stability of food prices and protect the vulnerable from variability that does occur; and tackle malnutrition” (16).

Again in a context of hunger, the proposal is to improve the access of the poor to technology to reduce inequalities, without discussing the control of technology or technological benchmarks; thus, helping oligopolies of the technology market to have more clients: “If these [modern] technologies are to make a useful contribution to the reduction of global hunger they have to both protect the environment and be accessible to farmers in low-income settings, otherwise inequities will persist and increase” (16).

In modern-colonial logic, it is essential to maintain epistemological hegemony. Those who do not exercise that hegemony must support it to benefit from it: “But to have a real effect, and to change the trajectory of planetary health, these local movements will need coherence, organization, and solidarity with the scientific and health communities” (16).

Those who exercise it have a voice and can be even more influential with the support of those who do not have voice: “The scientific and health communities, in turn, will be much more successful in influencing decision-makers who are feeling pressure for change from their constituents than they would without the support of civil society” (16).

In the medical care cost crisis, Private Medicine was clear and explicit in its intention to maintain the hegemony and avoid the participation of State Medicine (see the previous section). Similarly, the Rockefeller Commission is clear and explicit in its intention to maintain a top-down logic in which the owners of economic and scientific capital reserve for themselves the right to decide what is relevant: “Research funders and the academic community frame what questions get asked by scientists and can steer development of new ways of addressing major gaps in knowledge, scientific awareness, and academic focus” (16).

It is the modern logic imposed over centuries that says (note again the use of acceptable minimums and the meaning of acceptance): “At present trends, even with optimistic assumptions, the eradication of poverty (with a poverty line income of USD$5/day per person) will take 200 or 100 years for a poverty line of USD$1.25/day” (16). So there is no much to expect from modern trends.

It is necessary to overcome the modern crisis of civilization, starting from the first challenge identified in the report: “conceptual and empathy failures (imagination challenges)” (16).

## Ecology of Knowledge

The previous decolonial reading of the report, pointing to some of its possibilities, limits and obstacles, commits us from the global South to understand deeper causal levels and transform the current relationship between nature, health, and society. One possibility, not only alternative but above all critical, is the “ecology of knowledge” proposed by Santos, framed in what he calls “epistemologies of the South,” that is, the claim of the global South for “new processes of production, of valuing scientific and non-scientific valid knowledge, and of new relationships between different types of knowledge, based on the practices of the classes and social groups that have suffered, in a systematic way, destruction, oppression, and discrimination caused by capitalism, colonialism, and the naturalization of inequality” [translation is ours] (35).

According to Santos, non-Western forms of thought have been treated in an abyssal way by hegemonic modern Western thought, referring by abyssal to visible and invisible distinctions that divide social reality into two universes: one on this side of the line—the modern Western societies—and the other beyond the line—the colonial societies (35). For instance, in the field of modern knowledge, the visible line separates science from philosophy and theology, establishing the superiority of science through scientific criteria instead of reason or faith. The invisible line divides these types of knowledge from indigenous, popular, and other types of knowledge. The universe on the other side of the line disappears as reality. It becomes non-existent (in the sense of irrelevant and incomprehensible), radically excluded because it is beyond the universe of what the accepted conception of inclusion considers to be its other. In colonial societies, appropriation and violence segregate multispecies collectives, that is, subjects, nature, bodies, and knowledge that are on the side of denial (35).

Western modernity eliminates any reality that is on the other side of the line. Everything that does not fit in true-false or legal-illegal axes occurs in colonial zones (35). The abyssal lines are constitutive of the political and cultural relations based on the West and the interactions in the modern world-system (35). Thus, disqualification of non-modern knowledge globally underscores social and cognitive injustice.

By bringing these elements into the discussion about Planetary Health and One Health as alternatives for understanding and transforming the current relationship between nature, health, and society, the ecology of knowledge or post-abyssal thinking invites us to reflect and ask ourselves, among other things: if appropriation and violence established colonial societies, how can we now receive these philanthropic proposals under conditions of equality and justice instead of modernization imperatives? How to move toward a true post-abyssal thought?

Post-abyssal thinking takes the perspective of the other side of the line “precisely because the other side of the line has been the realm of the unthinkable in Western modernity” [translation is ours] (35). Post-abyssal thinking is learning from the epistemologies of the South, which confronts the “monoculture of modern science” against the ecology of knowledge. It frames science as one among many plural knowledge constituents, making possible a counter-hegemonic science to support marginalized multispecies collectives in their fight to get out of peripheries.

What is at stake is not only an abstract cognitive justice. The ecology of knowledge revalues the concrete interventions that different knowledge can offer (35). In it, knowledge hierarchies are context-dependent and not universal.

The ecology of knowledge invites us to build “an alternative of alternatives” based on permanent epistemological surveillance and intercultural translation. An alternative to avoid that Planetary Health, One Health, or any other approach become a renewed version of abyssal thinking, a softened revision of coloniality. From the ecology of knowledge we can stand against marginalizing apparatuses that create peripheries, unjust epidemiologic profiles, and only accept epistemologies of health from the global North.

## The Decolonial-colonial Axis in Which One Health of Peripheries must be Promoted

There are health-promoting indigenous lifestyles that serve as a reference to promote health in non-indigenous spaces. However, the adaptation of indigenous knowledge and experiences to non-indigenous peripheries leads to other types of practices (for instance, the Yanomami's respect and cultivation of edible mushrooms can inform agroecological practices but not simply scaled to supply the urban demand for such edibles). Not recognizing this transformation opens up colonizing possibilities that are counterproductive to health promotion. Globalization makes all locals contribute in some way to the reproduction of a colonial structure. Therefore, any place of decolonial resistance also has a colonial side, no matter how small. From this situation, one of the tasks for the decolonial promotion of One Health of Peripheries is to deconstruct, through the ecology of knowledge, the marginalizing apparatuses underlying health inequities suffered by multiple species (20). These are the issues addressed in this section.

In One Health of Peripheries, the peripheries are a symbolic category expressed in epidemiologic profiles (20). The global South is a heterogeneous geopolitical periphery within that category. Its health dimension has been theorized and transformed by Latin American Social Medicine since the 70s, and nowadays in the form of Critical Epidemiology, Collective Health, and South-South International Health. In a broader scope, this periphery, the global South, has promoted worldviews and lifestyles that in current rhetoric could be deemed sustainable, healthy, and instances of good living (36).

The indigenous worldviews and lifestyles, as well as the initiatives that have been based on them in the attempt to transform the institutional arrangement established and maintained by modernity, serve as a reference to promote One Health of Peripheries. Take good living (buen vivir) as an example, a concept from the Aimará *suma qamaña* and the Quechua *sumak kawsay*, incorporated in the constitutions of Bolivia and Ecuador (36). Although a discussion of good living is beyond the scope of this manuscript, we stress that in its generality it is a holistic proposal of collective-care exercised by a plural totality in which local communities are not peripheral (36). On the contrary, Planetary Health aims to control natural systems and keep the global South in a subaltern position. In it, the only allowed aspiration is to benefit from the epistemological, scientific, and technological transfers of the global North.

In institutional terms, the meaning of good living has been substantially transformed. Ecuador and Bolivia incorporated the concept in the constitution in 2008 and 2009, respectively, and just this by itself is a symbolic recognition of indigenous peoples. However, Solón point that in practice the recognized rights to nature and Mother Earth ended up being secondary to extractivist interests; the rhetoric of good living began to coexist with income redistribution policies that supported capitalist interests, allowed for the growth of oligopolies and encouraged patronage with some indigenous sectors (36). Paradoxically, under an indigenous government, it was possible to increase the acceptance of the modernization rejected for centuries, and the percentage of people who consider themselves indigenous fell from 62 to 41% between 1990 and 2013 (36). This experience of good living institutionalization shows that despite the marked differences between projects with opposite origins in the decolonial-colonial spectrum, the distance between discourses and implemented practices affects both poles of the spectrum. Contamination between the poles gives rise to the body of the spectrum.

The promotion of One Health of Peripheries must recognize and anticipate the distance between discourses and practices and the contamination between the decolonial and the colonial. Thus, it is convenient to consider the historical-social processes that produce and reproduce social organization levels and their corresponding epidemiological profiles. Following Samaja (37), individuals are in the lower social organization level, and the world-system is in the upper level. Between the two, there are several levels (family, community, political-administrative territorial divisions, contractual associations, and other institutions). Upper levels reproduce themselves by regulating the lower, but this regulation is not all-encompassing, allowing lower levels to produce partial changes in upper ones (37). The upper level reproduces a colonial structure through the regulation it exercises in lower levels, and these can partially change that structure through decolonial practices. This is the so-called social determination framing collective health epistemology, and as it has unavoidable multispecies dimensions, it also frames One Health of Peripheries (20). The promotion of One Health of Peripheries must occur in such dialectical movement, noting that partial decolonial changes means partial reproduction of coloniality. Such decolonial-colonial contradiction does not spare One Health of Peripheries, so proposals of promotion must take it into account to better match discourses and material possibilities.

The set of practices exercised from a given position has decolonial *and* colonial elements instead of decolonial *or* colonial elements. So indigenous good living and the neoliberal rhetoric of good living differ in the direction and intensity of bias toward the decolonial-colonial extremes. Similarly, collective health education programs are not totally different from colonial higher education or Preventive Medicine in its colonial origins. The degree of difference depends on how close they get to the respective extremes.

The conditions of possibility of the peripheral cartography (20) also condition the decolonial promotion of One Health of Peripheries. Exercising such promotion from within and outside that cartography challenges the center-periphery distinction through social determination movements. It is a utopian and dialectical promotion that, by centralizing peripheries, somehow reinforces the mentioned distinction and creates other peripheries. It is a *glocal* movement between localization and globalization (38).

So far, it may not be clear why it is convenient to add “One” to “Health of Peripheries.” It might well be Planetary Health of Peripheries to highlight the glocal movement between the global (planetary) and the local (peripheries). One Health is a conceptual framework that, like Planetary Health, brings together statements in favor of health for all, but in practice reinforces the myth of modernity. In fact, One World One Health™ is a registered trademark, created from the Wildlife Conservation Society conference, established in 2004 at Rockefeller University (39, 40). The colonial venue for the event may seem like an isolated event that does not link the Wildlife Conservation Society to coloniality. But suffice it to remember that at the time of Rockefeller institutions' foundation, the Bronx Zoo was exhibiting Ota Benga (the young Mbuti from what is now the Democratic Republic of the Congo). The Wildlife Conservation Society waited until 2020 to issue a public apology for its responsibility in the exhibition and the position of two of its founders, Madison Grant and Henry Fairfield Osborn, who were also founders of the American Eugenics Society and stood in favor of defendants in the Nuremberg trials (41). Unfortunately, the apology did not entirely reproach the colonial tradition of exhibiting other animals, perhaps mistaking exhibition as a necessary condition for wildlife conservation. They did not see anything wrong with exhibiting Ota Benga a century ago, and now they do not condemn the same practice with nonhuman animals. Hopefully, they will not need another century to abolish that practice.

In light of the colonial roots of One Health, which goes beyond what we briefly outlined (18), a decolonial proposal based on the One Health concept may seem contradictory. However, it is worth noting that One Health of Peripheries gives other meanings to One Health (20) and metabolizes contradictions through its social determination and the ecology of knowledge. Decolonizing One Health adds plurality to the Latin American health movements, thus increasing the strength and resilience of decolonial resistance.

In One Health, health is more-than-human, and it involves three inextricably related domains: human health, animal health, and environmental health (42). This differs from Planetary Health in which health is human and natural systems have instrumental value as determinants of health (16); the value of animals is instrumental to the extent that they contribute to the maintenance of natural systems favorable to human health. In One Health approaches, animals are also predominantly instrumental to human health (42); however, they appear as carriers of health, and animal health takes a fundamental role in a health that is not just human.

Biomedicine does not question the existence of physiopathological processes in animals in the same way that epidemiology does not question the existence of transmission dynamics between animals or between animals and humans. As any other species, humans have similarities and differences with the individual and population biological processes of health-disease of other species.

The attribution of lower moral status to non-human animals for the simple fact of not belonging to the human species (speciesism) is as arbitrary as giving less value to some humans because of the race or gender attributes tied to them [racism and sexism] (43). Attempts to justify the inferior status of animals sometimes base arguments on the greater cognitive capacity of humans. However, many animals surpass the cognition of severely disabled humans, leading to justifications of moral differentiation in which not all humans are of equal value and some are of less value than many animals (43). Based on different criteria of cognitive capacity, the moral justifications to completely separate human beings from the rest of the animals are also problematic, revealing what Agamben calls the anthropological machine, an inclusion-exclusion apparatus to separate humans from other animals, that the more it is renewed in the attempt to eliminate aporias, the more it reveals its arbitrariness and contradiction (44).

The distinction between humans and non-humans is a marginalizing apparatus in the service of domination. It is a central dichotomy of modernity (45) through which dehumanization/animalization is all the more, the greater the distance of a being from the Western heterosexual male referent. It is epistemic violence that marginalizes humans, denies the subjectivity of other animals, and reconfigures animality as black, indigenous (45), female, and not heterosexual. In other words, it is more than human violence, with victims of multiple species. Animals are animalized insofar as they are inscribed in such animal space of colonial domination (45). The animalizing apparatus is also applied through colonial health practices that legitimize domination and represent it as a benevolent act.

In his analysis of 19th-century slave farms in Cuba, Camacho describes how Chateusalins, in his Vademecum of Cuban landowners, recommended masters of female slaves “to avoid giving them a harsh treatment,” to give them “better food than before” and to “protect them with delicacies and concessions to encourage them to preserve the product of their conception and raise their little offspring” [translation is ours] (46). In order to convince the masters, Chateusalins stated: “I know that in all farms where it reigns goodness and sweetness and attentions of the masters toward the blacks, there are many happy blacks whose mothers express their happiness in their singing and smiling faces [...] We have seen the books of gains and losses in which it appears that far from suffering a loss of 5.5%, which is what is generally calculated in this class of farms, it has been, on the contrary, an increase from 4.5 to 5.5%, which shows the advantages that the careful treatment given to blacks brings with it” [translations is ours] (46).

This production-health binomial was framed in what we might understand as an epidemiological-zootechnical approach for slave control. Compartmentalization of facilities; populations divided according to demographic criteria of productive and reproductive interest; classification and monitoring of morbidity and mortality; prevention of communicable diseases; reproductive selection (genetic improvement); hygiene, nutrition, socialization, and other generic practices to reduce losses of biological capital [see the documented analyzes of such practices by Smithers and Camacho (46, 47)].

The rationalizing discourse of such an approach—statistics, efficiency, evidence—sought above all productivity, adding value to animalized commodities. The slaves were objects of knowledge and professional practices (medicine, statistics, anthropology) that produced “truths” on which the political and economic regime of the plantation depended (46). However, behind the pretense of truth and rationality, there was prejudice and contradiction. As shown by the Camacho's analysis of the medical anthropology of Dumont (48), the medical literature provided descriptions of the black race as “prone to contracting several diseases,” while the anthropological one contributed with assertions of the type “lazy by nature,” “all blacks are polygamous,” “all are fetishists” [translations are ours] (46). On the other hand, the prescriptions of kindness and attention to the “human” needs of the slaves ironically opposed animalization, but this did not prevent its practice. The concern with the health of the slaves was a concern to maintain the profitability of their bodies and prevent them from transmitting diseases to the masters and their families, whose health did have value in itself. The epidemiology and zootechnics of slaves coexisted with torture practices to make them docile; their affections were irrelevant, except as instruments to increase productive and reproductive performance, through persuasive practices also reported in the medical literature (46). The advertisements of slaves with specific phenotypic characteristics and of drugs authorized by the government against diseases affecting slaves (46) showed how animalization was naturalized and legitimized by the State, the media, and the knowledge produced by epistemic authorities.

In essence, the discourse of slaves epidemiology and zootechnics is equivalent to the contemporary discourse of animal production epidemiology and animal science. Similarly, within animal welfare science we find benevolence narratives that legitimize livestock exploitation and add value to live commodities. In both cases (slaves and livestock), oppressive relationships are naturalized, and the better performance of productive and sanitary parameters serves as an indicator of improvements in well-being.

By deconstructing marginalizing apparatuses and giving rise to multispecies collectives in which the other is not a commodity and its subjectivity is cared for and respected, the possibilities of promotion cease to be variations of degree within a restrictive peripheral space and become variations of kind. Thus, abolishing slavery is a leap of promotion, allowing lifestyles—processes, capabilities, and health conditions—unattainable through the health practices restricted to the periphery of slavery. As health is inherently determined by value judgments, problematizing these judgments is essential to break the margins that limit the promotion of One Health of Peripheries.

## Seven Actions of Decolonial Promotion of One Health of Peripheries

The Ottawa Charter [see in McPhail-Bell et al. (49) a postcolonial critique of the Charter] proposed five actions to promote health: (1) build healthy public policy; (2) create supportive environments; (3) strengthen community actions; (4) develop personal skills, and; (5) reorient health services (50). Redefining and complementing these actions with another two lead to the promotion of One Health of Peripheries: (1) deconstruct marginalizing apparatuses; (2) enrich the ecology of knowledge; (3) build healthy multispecies public policy; (4) create supportive environments; (5) strengthen multispecies community actions; (6) develop individual capabilities in multiple species, and; (7) reorient multispecies health services. The deconstruction of marginalizing apparatuses is transversal to the other actions, and in that sense, we do not need to include it as a separate action. However, we can do the same with the others. Although one is transversal to the others, its explicit recognition reinforces its importance.

The seven actions require overcoming the primary challenge identified by the Lancet Commission on Planetary Health: “conceptual and empathy failures (imagination challenges)” (16), something particularly challenging within coloniality. However, for the very same reason, they contribute to the decolonial turn. A turn that requires imagination and multispecies empathy, and might be seen as a turn from Capitalocene to Chthulocene [see in Haraway (51) a discussion of the Anthropocene, Capitalocene, and Chthulocene].

Despite the difficulties in promoting One Health of Peripheries, there are precedents for each of its seven actions. The first action deconstructs marginalization from a health perspective and finds support in the more-than-human sociology (52–55), anthropology (56), biopolitics (44, 57, 58), critical studies (59), social work (60, 61), theories justice (62–64), and moral philosophy (65, 66) to name a few areas. The second action opens space to the epistemologies of the indigenous and non-indigenous global South (5), remembering that for the holistic sustainability “discovered” by Planetary Health, there are indigenous versions with centuries of successful experiences and that animalization is a colonial apparatus that oppresses human and non-human animals. Albeit insufficient, there is already public policy support for living cities (67), biodiversity and indigenous territories. More-than-human theories of labor (63), food sovereignty and security, sustainable agriculture, response to disasters, and degrowth perspectives can strengthen and expand this type of policies (third and fourth action). Moreover, participatory policies exist in various settings, community practices abound in the global South, and animal and environmental activism has been growing. This gives practical support to multispecies intersectionality (20, 68), from which the fifth action can be worked out. An outstanding theoretical framework of justice is that of capabilities, already elaborated by Nussbaum to consider disability, nationality, and non-human animal species (62). Therefore, the sixth action, which in the version of the Ottawa Charter (fourth action) might seem applicable only to humans, has a robust theoretical support to consider peripheral subjects of different species. Even the seventh action has precedents. In Brazil, for instance, the Unified Health System (national health system), in addition to having units dedicated to the epidemiological surveillance of zoonoses that also promote responsible care for animals, has dependencies dedicated to the health and protection of domestic animals. These dependencies have specific attributions regarding rescuing, sheltering and adoption, population control, and administration of veterinary hospitals offering free services (69). Undoubtedly, some of these precedents need reassessments and sound plural participation to preclude or stop being stratagems at the service of non-collective interests. But at the same time, they are precedents that in some way have locally fractured peripheries-making margins.

## Conclusion

Coloniality did not end with colonialism, and the myth of modernity is at the kernel of the crisis of civilization we are living. Philanthrocapitalism allows material gains that significantly improve the livelihood of the poorest because they are in conditions in which small aids make a big difference, even if they continue in poverty. Those improvements are convenient to legitimize vast accumulations of wealth by a few rich philanthropists and massive deprivation suffered by billions (in 2019, the wealth concentrated by 0.000028% of the population was greater than that of 59.7%). Philanthrocapitalism in health has been a strategy to reinforce colonial epistemology and favor the interests of the global North, dictating what should be understood by health, how health problems should be solved, and how people should live to avoid them. The Rockefeller Foundation has been an icon of philanthrocapitalism, shaping Latin American health through public policy, education, and research. One of the Foundation's recent proposals is Planetary Health, also framed in the rhetoric of the global North.

The ecology of knowledge, with its intercultural translation, is a response from the global South to repair the damage of coloniality. It encompasses indigenous and popular knowledge, Latin American health movements, and the counter-hegemonic use of science. It can also make counter-hegemonic use of Planetary Health and One Health. An example of such use is One Health of Peripheries, at the same time a reconfiguration of One Health and Latin American health movements, strongly opposed animalization, that is to say, to the colonial space oppressing animals and peripheral human groups. Extending the scope and the meaning of the Ottawa Charter proposal, the decolonial promotion of One Health of Peripheries comprise seven actions: (1) deconstruct apparatuses of marginalization; (2) enrich the ecology of knowledge; (3) build healthy multispecies public policy; (4) create supportive environments; (5) strengthen multispecies community actions; (6) develop individual capabilities in multiple species, and; (7) reorient multispecies health services.

## Data Availability Statement

The original contributions generated for the study are included in the article/supplementary material, further inquiries can be directed to the corresponding authors.

## Author Contributions

All authors contributed to the article and approved the submitted version.

## Conflict of Interest

The authors declare that the research was conducted in the absence of any commercial or financial relationships that could be construed as a potential conflict of interest.
